# The biology of YAP in programmed cell death

**DOI:** 10.1186/s40364-022-00365-5

**Published:** 2022-05-23

**Authors:** Yifan Cheng, Misha Mao, Yong Lu

**Affiliations:** 1 Department of Gastrointestinal Surgery, Taizhou Hospital of Zhejiang Province, Wenzhou Medical University, Taizhou, Zhejiang China; 2 Department of Surgical Oncology, Sir Run Run Shaw Hospital, Zhejiang University, Hangzhou, Zhejiang China

**Keywords:** YAP, Programmed cell death, Apoptosis, Autophagy, Ferroptosis, Pyroptosis

## Abstract

In the last few decades, YAP has been shown to be critical in regulating tumor progression. YAP activity can be regulated by many kinase cascade pathways and proteins through phosphorylation and promotion of cytoplasmic localization. Other factors can also affect YAP activity by modulating its binding to different transcription factors (TFs). Programmed cell death (PCD) is a genetically controlled suicide process present with the scope of eliminating cells unnecessary or detrimental for the proper development of the organism. In some specific states, PCD is activated and facilitates the selective elimination of certain types of tumor cells. As a candidate oncogene correlates with many regulatory factors, YAP can inhibit or induce different forms of PCD, including apoptosis, autophagy, ferroptosis and pyroptosis. Furthermore, YAP may act as a bridge between different forms of PCD, eventually leading to different outcomes regarding tumor development. Researches on YAP and PCD may benefit the future development of novel treatment strategies for some diseases. Therefore, in this review, we provide a general overview of the cellular functions of YAP and the relationship between YAP and PCD.

## Background

The transcriptional coactivator Yes-associated protein (YAP) is amplified in human cancer and acts as an oncogene in various cancers [1–3]; its abundance and activity are increased in many types of cancer, including gastric cancer, colorectal cancer, liver cancer and breast cancer, in which it plays an important role in initiation, progression, metastasis, and drug resistance [4–7]. YAP and its paralog transcriptional coactivator with PDZ-binding motif (TAZ) are the major downstream effectors of the Hippo pathway, which controls organ size and cell fate during embryonic development [8]. Phosphorylated YAP can be sequestered in the cytoplasm and then degraded by the ubiquitin–proteasome system. Unphosphorylated YAP can enter liquid droplets and then translocate to the nucleus, where it has a series of functions [7, 9]. Nuclear YAP participates in complex and only partially understood molecular cascades that are responsible for the oncogenic responses by regulating multiple processes, in addition to the abovementioned processes, and by acting as an important mediator of cancer immunity [10] and cancer cell interactions [11].

It has also been discovered that YAP plays an important role in the regulation of programmed cell death (PCD) in cancer [12–14]. Different forms of PCD, including apoptosis, autophagy, ferroptosis and pyroptosis, have been characterized [15–19]. Understanding the connection between YAP and PCD will promote the development of more effective strategies for the treatment of some diseases, especially cancer. In this review, we discuss the regulation of YAP and the connections between YAP and PCD.

### Synthesis and regulatory system of YAP

**Fig. 1:**
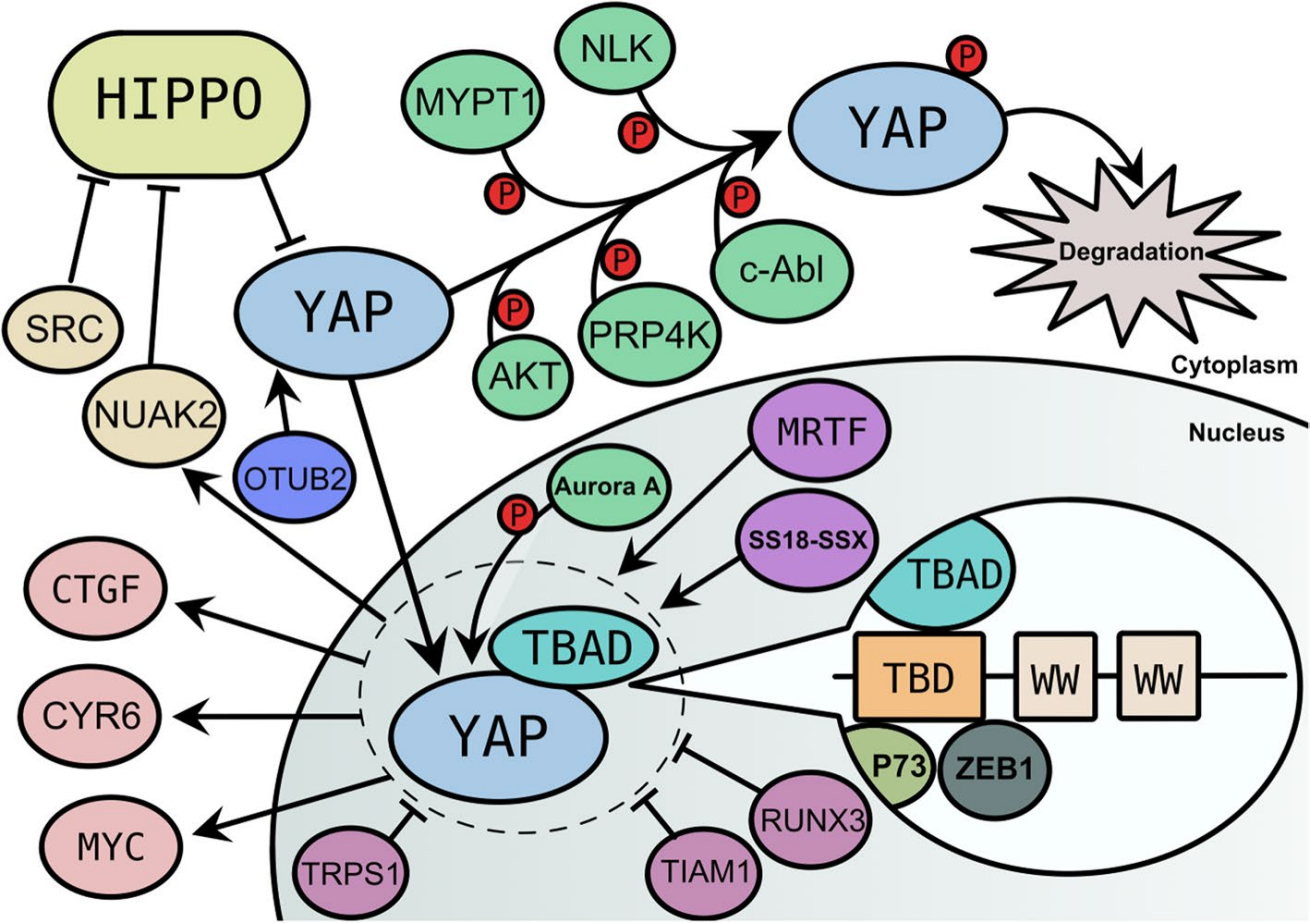
Signaling pathways and genes controlling YAP expression and its regulatory system. YAP can be affected by many factors. when YAP is phosphorylated in the cytoplasm by some factors like Hippo pathway, AKT, c-Abl, NLK, MYPT1 and PRP4K, it finally can be degraded, YAP can also be enhanced by OTUB2 so that it can enter in nucleus easily. Active YAP localizes in the nucleus and combines with TEAD, p73 or ZEB1 at its TED domain thus affect other factors. YAP-TEAD co-activator could enhance CTGF, CYR6, MYC and NUAK2, which participates in a feed-forward loop that enhances YAP activity by inhibit Hippo pathway. YAP-TEAD co-activator could also be inhibited by TRPS1, TIAM1 and RUNX3, and be enhanced by MRTF and SS18-SSX. Aurora a kinase can phosphorylate YAP in nucleus thus reduce the activity of it

YAP, a small 350 kb amplicon encoding a 65 kDa protein on human chromosome 11q22, was identified in a screen for gene copy number alterations in mouse mammary tumors [20, 21]. YAP protein, which is highly conserved across different species, has a N-terminal TEAD-binding domain (TBD) followed by one or two WW domains and a C-terminal transactivation domain [22–24]. The WW domain is a protein–protein interaction module with two signature tryptophan residues spaced 38 to 40 amino acids apart [22, 25, 26]. However, as YAP lacks a DNA-binding motif, it regulates gene transcription by binding transcription factors (TFs) such as TEAD1–4, p73 and ZEB1 through its TBD and WW domains [27–37]. The YAP–TEAD complex regulates the expression of many oncogenes (Fig. 1), such as CTGF, CYR61, MYC and NUAK2 [28, 38–40]. NUAK2 participates in a feed-forward loop that enhances YAP activity [38]. In addition, YAP–TEAD transcriptional activity can be regulated by different proteins through many mechanisms. For example, the SS18-SSX fusion protein and MRTF potentiate TEAD-YAP activity [41, 42], whereas TIAM1 and RUNX3 reduce YAP–TEAD transcriptional activity [31, 43].

On the other hand, the translocation of YAP between the nucleus and cytoplasm has emerged as an important means of regulating YAP activity [34, 44, 45]. Active YAP localizes in the nucleus. YAP can be negatively regulated by its circular RNA circYAP, which suppresses the assembly of translation initiation machinery [46]. In addition, YAP protein activity can be modulated by posttranslational modification. OTUB2 activates YAP protein by direct deubiquitination and stabilization [47]. The Hippo signaling pathway is the main regulator of YAP activity, which is accomplished by modulating YAP phosphorylation [48–52]. Through this signaling pathway, YAP can be phosphorylated at five conserved HXRXXS motifs, including the serine 127 (S127) and S397 sites [51]. Additionally, other pathways can regulate YAP activity though Hippo pathway. For instance, MYPT1 activates the Hippo pathway, resulting in YAP suppression [53]. SRC phosphorylates Hippo pathway kinases, thus promoting YAP activity [54]. PRP4K phosphorylates YAP at S111 and S250 to regulate this protein in parallel with the Hippo pathway [55]. The STARD13-correlated ceRNA network leads to the nuclear–cytoplasmic translocation of YAP through two independent pathways, the Hippo pathway and Rho-GTPase/F-actin signaling [56]. AKT and c-Abl phosphorylate YAP at S127 and Y357, respectively, thus suppressing some transcriptional responses [57, 58]. NLK phosphorylates YAP at S128 to evoke YAP redistribution into the nucleus [59]. YAP interacts with Aurora A kinase in the nucleus, which leads to specific phosphorylation of YAP, and then potentiates YAP-mediated transforming ability [60]. Therefore, in the future, it will be important to examine the contribution of different TFs to the regulation of YAP phosphorylation, nuclear localization and to determine the underlying mechanism in greater detail. Meanwhile, the effector molecules downstream of YAP should also be identified.

### YAP and PCD

PCD is a normal physiological phenomenon. Under normal circumstances, PCD can help eliminate senescent and dangerous cells, such as cancer cells and infected cells. However, dysregulation of cell death often has negative consequences. And there is a crosstalk among the four major forms of PCD, apoptosis, autophagy, ferroptosis and pyroptosis [15–19]. PCD is typically dysregulated in certain diseases, especially in cancer, and this dysregulation provides necessary conditions for tumor progression. Many oncogenes and tumor suppressor genes are linked to tumorigenesis through PCD, and some proteins and RNAs can simultaneously regulate these different forms of cancer cell death [61]. Previous research has identified YAP-related regulatory networks in cancer. Among these networks and pathways, the evolutionarily conserved Hippo pathway plays an important role in cell growth and organ size control during embryonic development. As the center of this complex network, YAP is necessary for PCD controlled by the Hippo pathway, but YAP is also regulated by many other factors and regulates additional factors to affect PCD (Table 1); these findings prompts researchers to further investigate the relationship between YAP and PCD.

**Table 1:** The relationship between the changes of YAP and various types of PCD in different cells

**Cell types**	**Upstream**	**YAP**	**Downstream**	**Pathway**	**Non-genetic contexts**	**PCD**	**Citation**
**Hepatocellular cancer**		YAP-TEAD	Jag-1↑	Notch signaling		Anti-Apoptosis	[62]
**Breast cancer**		YAP-TEAD	BCAR4↑	Hedgehog signaling		Anti-Apoptosis	[63]
**Hepatocellular cancer**	Fbxw7	YAP-TEAD↓		Proteasomal degradation		Apoptosis	[64]
**Urothelial cell cancer**		YAP-TEAD↓			DNA-damage	Apoptosis	[65]
**Breast cancer**	Akt	YAP-p73↓		Pro-apoptosis genes↓		Anti-Apoptosis	[57]
**Colon cancer** **Breast cancer**	c-Abl	YAP-p73↑		Pro-apoptosis genes↑	DNA-damage	Apoptosis	[58]
**Cholangiocarcinoma**		YAP-TEAD	MFAP5↑	Promotes angiogenesis		Anti-Apoptosis	[66]
**Colorectal cancer**	GPRC5A	YAP↑		Anti-apoptotic genes↑	Hypoxia	Anti-Apoptosis	[67]
**Breast cancer**		YAP-TEAD↑		Autolysosome degradation	Nutrient deprivation	Autophagy	[68]
**Hepatocellular cancer**		YAP↑		RAC1-ROS-mTOR pathway	Multi-drug resistance	Anti-Autophagy	[69]
**Colorectal cancer**		YAP-TEAD	Bcl-2↑			Anti-Autophagy	[70]
**Glioblastoma**		YAP	HMGB1↑			Autophagy	[71]
**Epithelial cell**	NF2	YAP-TEAD↓		Hippo pathway	High cell density	Sensitivity of Ferroptosis↓	[72]

### YAP and apoptosis

Apoptosis is initiated in cells in response to diverse physiological and pathological stimuli that ultimately leads to the activation of apoptotic pathways [73]. The dysregulation of apoptosis is one of the classical hallmarks of the maintenance and regulation of tumor growth [74–76]. YAP shows antiapoptotic effects in various cells; for example, YAP reduces apoptosis by upregulating Jag-1 to activate Notch signaling in hepatocellular carcinoma cells, and enhancing BCAR4 expression in breast cancer, respectively [62, 63]. In addition, the ubiquitination of YAP by Fbxw7 and its subsequent proteasomal degradation enhance apoptosis in hepatocellular carcinoma cells, while restoring YAP expression partially abrogates Fbxw7-induced cell apoptosis and growth arrest in vitro and in vivo [64]. YAP also enhances the ability of cancer cells to resist apoptosis induced by chemotherapy. Hepatocellular carcinoma patients with a poor response to transarterial chemoembolization (TACE) had higher YAP protein levels than responsive patients, indicating that YAP enhances the chemoresistance of Hepatocellular carcinoma by blocking apoptosis [5]. Additionally, Ciamporcero et al. found that YAP knockdown sensitizes urothelial carcinoma cells to chemotherapy and radiation via the increased accumulation of DNA damage and apoptosis [65]. These studies show that the overproduction of YAP can enhance the resistance of cancer cells to apoptosis, and when YAP activity is inhibited, the increase in apoptosis sensitizes cancer cells to chemotherapy.

In cholangiocarcinoma, YAP and TEADs prevent apoptosis induced by cytotoxic drugs together, but YAP knockdown sensitizes cholangiocarcinoma cells to drug-induced apoptosis [66]. Related research on hypoxic cancer cells showed that YAP can be activated by GPRC5A, a novel hypoxia-induced protein that protects cancer cells from apoptosis during oxygen deprivation, leading to the downregulation of proapoptotic target genes and the increased survival of hypoxic cancer cells. This study further showed that the apoptosis induced by GPRC5A depletion under hypoxic conditions could be rescued by constitutively active YAP [67]. YAP prevents apoptosis in acute pancreatitis, possibly by modulating the inflammatory response [77]. Taken together, the data indicate that YAP acts as a core factor in regulating cancer apoptosis through various mechanisms, and in the presence of apoptotic stimuli, YAP seems to enhance the ability of cells to mitigate apoptosis and survive.

Interestingly, YAP can also function as a proapoptotic factor. When cells experience stress due to severe DNA damage, Akt and c-Abl phosphorylate YAP, thus suppressing the induction of proapoptotic gene expression and ultimately inducing apoptosis [57, 58]. YAP can trigger apoptosis by binding p73 instead of TEAD and thereby upregulating the proapoptotic gene BAX [78–80]. These data contradict the reported function of YAP as an oncogene, and it remains unknown whether targets of the YAP–p73 complex other than BAX are relevant to promoting apoptosis and whether the engagement of other TFs by YAP can elicit a proapoptotic transcriptional program. Elucidation of the precise mechanisms by which YAP modulates apoptosis might yield new therapeutic targets.

### YAP and autophagy

Autophagy is a process by which intracellular damaged or excessive organelles and cytoplasmic proteins are degraded through a lysosome-mediated process [81–83]. This evolutionarily conserved process enables the recycling of nonessential cytosolic materials to maintain cellular homeostasis and overcome metabolic stress and nutrient deprivation. A close association between autophagy and cancer has been established, and autophagy plays an important role in cell survival and death [82, 84–87]. Excessive or prolonged autophagy can elicit cell death. Some studies showed that YAP is regulated by autophagy and thus affects cell fate [88–91]. Impaired autophagy is a driver of nuclear YAP localization and promotes YAP activity to enhance tissue remodeling and carcinogenesis in hepatocellular carcinoma cells [92, 93]. Conversely, triple-negative breast cancer utilizes autophagy as a mechanism to promote YAP nuclear entry, thus promoting cell invasion and metastasis [94]. As mentioned above, YAP requires autophagy to sustain the transformed characteristics of cancer cells. And YAP is also involved in the lysosomal degradation of autophagosomes and the fusion of autophagosomes with lysosomes [95]. Therefore, YAP is both upstream and downstream of autophagy. YAP increases autolysosome degradation, thereby enhancing cellular autophagic flux to protect breast cancer cells from nutrient deprivation-induced apoptosis [68]. YAP upregulation endows hepatocellular carcinoma cells with multi-drug resistance via the RAC1–ROS–mTOR pathway, resulting in the repression of autophagy [69]. YAP can inhibit autophagy in human colorectal cancer cells by transcriptionally upregulating Bcl-2, which consequently promotes colorectal cancer progression [70]. Zhao et al. found that in glioblastoma, YAP enhances HMGB1-mediated autophagy to promote glioma progression [71]. The relation between YAP and autophagy is not consistent, as it depends on other factors. Pavel et al. found that the response of YAP to autophagy can differ due to α-catenin levels: high basal α-catenin levels enable YAP to positively regulate autophagy, while low α-catenin levels lead to a negative correlation between YAP and autophagy [96]. Since autophagy can provide nutrients to cells and remove toxic particles autonomously, this biological process is critical for cell survival, especially under nutrient-deprived conditions, such as in the tumor microenvironment. YAP-related autophagy seems to be the first step in inducing pathways and processes that prevent cancer cells from dying [68]. In the future, autophagy-related death may be controlled by regulating YAP to prevent tumor progression, The mechanism underlying the relationship between YAP and autophagy needs to be explored further.

### YAP and ferroptosis

Ferroptosis is distinct from the above forms of cell death; since it was first proposed in 2012, it has gradually become a widely researched topic [97–99]. Ferroptosis, which is driven by cellular metabolism and iron-dependent lipid peroxidation, has been implicated in cancer growth and drug resistance [100–103]. Epithelial-to-mesenchymal transition (EMT) is believed to generate cancer stem cells, resulting in metastatic spread and contributing to clinical therapeutic resistance [104]. YAP was recently reported to be necessary for promoting EMT [105, 106]; however, it has been confirmed that EMT can improve the sensitivity of cells to ferroptosis [107, 108], perhaps indicating that YAP promotes ferroptosis through EMT. In later studies, researchers found that the sensitivity to ferroptosis is highly influenced by cell contacts and cellular density [109]. The activation of TFs in the Hippo pathway (such as YAP and TAZ) promotes ferroptosis in cancer cells by regulating the expression of ferroptosis modulators, such as ACSL4, TFRC, EMP1 and ANGPTL4 [72]. In addition, ovarian and renal cancer cells were found to be sensitive to ferroptosis induced by erastin treatment and cystine deprivation when they grew at low density or as individual cells. However, when these same ferroptosis-sensitive cells were grown to confluence or in a high-density situation (like three-dimensional (3D) spheres), they become highly resistant to ferroptosis [110]. As a sensor of cell density, TAZ can regulate cellular sensitivity to ferroptosis through the Hippo pathway in ovarian cancer and renal cell carcinoma, instead of YAP [109, 111]. Wu et al. reported an explanation for this phenomenon. In epithelial cells and mesothelioma, cell–cell contacts inhibit ferroptosis partly through the cadherin 1-mediated inhibition of YAP transcriptional activity. Cells at high density had high levels of ECAD, which activates Merlin and subsequently regulates YAP activity by activating the Hippo pathway, thus influencing the sensitivity to ferroptosis. In addition, HCT116 cells lacking YAP were no longer sensitized to ferroptosis by RNAi-mediated Merlin knockdown, demonstrating that Merlin suppresses ferroptosis by inhibiting YAP activity [72]. There have been few studies on YAP and ferroptosis, the mechanism underlying the relationship between YAP and ferroptosis needs to be explored further. In addition, autophagy is closely related to ferroptosis, and YAP is an important regulator of autophagy. The relationship between these two processes and YAP will be discussed below.

### YAP and pyroptosis

Pyroptosis, also known as inflammatory necrosis, is a kind of PCD characterized by the continuous expansion of cells until the cell membrane ruptures, leading to the release of cell contents, which activate a strong inflammatory response. YAP expression is higher in triple-negative breast cancer than in other types of breast cancer, and YAP promotes the metastasis of triple-negative breast cancer cells [112–114]. Studies have also found that PD-L1 expression is higher in triple-negative breast cancer than in other types of breast cancer [115, 116], and studies in other types of cancer have identified mutual regulation between PD-L1 and YAP [117]. For example, in BRAF inhibitor-resistant melanoma cells, YAP induces the expression of PD-L1, which participates in tumor escape [118]. Researchers have found that under hypoxia, PD-L1 accumulates in the nucleus, whereby it can regulate the expression and activation of gasdermin C, the main executor of pyroptosis, to promote this form of cell death [119]. Therefore, YAP may regulate pyroptosis by regulating various factors at the protein and mRNA levels. In macrophages, flagellin secreted by Salmonella activates the Nod-like receptor family CARD domain-containing protein 4 (NLRC4) inflammasome, leading to the production of IL-1β and pyroptosis of infected cells. In contrast, in Salmonella-infected B cells, infection induces YAP phosphorylation and promotes the interaction of YAP with Hck, thus preventing the transcriptional activation of NLRC4 and blocking B cell death. Although the outcomes of cellular Salmonella infection are variable, YAP activation can lead to pyroptosis [120]. Cui et al. found that in pancreatic ductal adenocarcinoma, MST1 could suppress the proliferation, migration, invasion, and spheroid formation of pancreatic ductal adenocarcinoma cells via caspase 1-induced pyroptosis mediated by reactive oxygen species (ROS) [121]. Whether MST1, a core component of the Hippo pathway, can influence pyroptosis by affecting YAP activation is worth studying.

### Crosstalk between YAP and PCD

There are also various connections between different types of PCD. Although the forms of PCD differ from each other in the mechanism of death, they are related. For example, autophagy is necessary during ferroptosis. As a selective form of autophagy, ferroptosis is initiated by the degradation of ferritin, which triggers labile iron overload, lipid peroxidation, membrane damage, and cell death [122]. Yang et al. discovered a type of autophagy called “clockophagy”, which involves the selective degradation of the core circadian clock protein ARNTL and can facilitate the induction of ferroptosis [123]. Gao et al. identified multiple autophagy-related genes as positive regulators of ferroptosis and showed that ferroptosis is an autophagic cell death process [124]. Thus, ferroptosis and autophagy are closely linked. Furthermore, autophagy enhances labile iron availability and potentiates the activity of ferroptotic stimuli in glutamic-oxaloacetic transaminase 1(GOT1)-knockdown cells, as GOT1 inhibition represses mitochondrial metabolism and promotes a catabolic state [125]. Meanwhile, research on the dependence of breast cancer cells on exogenous glutamine for growth showed that YAP can induce GOT1 [126]. These data indicated that YAP could weaken the link between autophagy and ferroptosis, thereby protecting cells. Taken together, the evidence suggests that YAP plays an important role in the link between autophagy and ferroptosis.

Recent studies have revealed that autophagy can facilitate apoptosis in different ways. Autophagy promotes apoptosis through the degradation of antiapoptotic and cell survival factors; for instance, the autophagic degradation of caveolin-1 contributes to apoptosis in rat hippocampal astrocytes [127]. The product of the autophagy-related gene Atg5 is required for the formation of autophagosomes. The calpain-mediated cleavage of Atg5 generates a proapoptotic protein that associates with the antiapoptotic molecule Bcl-xL to stimulate intrinsic apoptosis [128]. However, the presence of YAP alters this relationship; in particular, YAP-induced autophagy always inhibits apoptosis to elicit cancer cell survival. In an immunohistochemical analysis of clinical thyroid papillary carcinoma tissue microarrays, YAP silencing was accompanied by decreases in Beclin1 and Atg5 expression [129]. In addition, YAP prompted an increase in autophagy by enhancing autolysosome degradation; thus, YAP plays a protective role in cisplatin-resistant human ovarian cancer cells by reducing apoptosis [130]. When breast cancer cells are exposed to nutrient deprivation, YAP increases autolysosome degradation, thereby enhancing autophagic flux in breast cancer cells and protecting these cells from apoptosis [68]. As stated above, the relationship between YAP and autophagy is complex, but YAP ultimately regulate autophagy and then affect cell survival by inhibiting other forms of PCD.

Apoptosis is also connected to ferroptosis. In a study of acute lung injury (ALI), ferroptosis contributed to intestinal ischemia/reperfusion-induced ALI in vivo, and inhibitor of apoptosis-stimulating protein of p53 (iASPP) was found to inhibit ferroptosis and alleviate intestinal ischemia/reperfusion-induced ALI through the Nrf2/HIF-1/TF signaling pathway [131]. In a study on bladder cancer, researchers demonstrated the presence of cross-talk between YAP and Nrf2 [132]; therefore, although there is not sufficient evidence to prove its role in the relationship between apoptosis and ferroptosis, YAP is very likely involved.

YAP can prevent further apoptosis by regulating the inflammatory response [77], with which pyroptosis is strongly associated [133]; these data suggest that YAP can connect these two types of PCD. However, there is a lack of research in this area. In summary, YAP plays a key role in the regulation of various forms of PCD and their relationships, and the specific mechanism is not yet clear and worthy of further in-depth study. In addition, researchers need to focus on the involvement of YAP in the relationships among different types of PCD.

## Conclusion

Since the discovery of YAP several decades ago, the biological functions and consequences of YAP have gradually been revealed. YAP is an important downstream effector of the Hippo pathway and a transcriptional cofactor involved in the transcription of many known important tumor factors. As such, YAP is an important factor that should be studied in relation to tumorigenesis, including tumor development and metastasis, as well as the prevention of drug resistance. Numerous studies have shown that YAP is involved in cancer cell death. Our review summarizes the knowledge of PCD and YAP, explores the important regulatory role of YAP in PCD. As stated above, YAP not only regulates individual types of PCD but also is likely a bridging factor that connects different types of PCD. As an oncogene, YAP is supposed to promote cancer progression and inhibit PCD, however, YAP is found to promote PCD such as apoptosis [134] and ferroptosis [72] in some studies. There is great value in discovering the specific underlying mechanism. Moreover, the role of YAP in pyroptosis remains unclear but will be further explored in future research. Research on the association between YAP and PCD could help to establish a systematic tumorigenesis mechanism, guide the development of new cancer treatments, and aid in the discovery of treatments for other inflammatory-related diseases.

## Data Availability

Not applicable.
